# The Microbiota Determines Susceptibility to Experimental Autoimmune Uveoretinitis

**DOI:** 10.1155/2016/5065703

**Published:** 2016-05-17

**Authors:** Jarmila Heissigerova, Petra Seidler Stangova, Aneta Klimova, Petra Svozilkova, Tomas Hrncir, Renata Stepankova, Miloslav Kverka, Helena Tlaskalova-Hogenova, John V. Forrester

**Affiliations:** ^1^Department of Ophthalmology, First Faculty of Medicine, Charles University in Prague and General University Hospital in Prague, U Nemocnice 2, 12808 Prague 2, Czech Republic; ^2^Institute of Microbiology of the Czech Academy of Sciences, v.v.i., Prague, Videnska 1083, 14220 Prague 4, Czech Republic; ^3^Institute of Experimental Medicine of the Czech Academy of Sciences, v.v.i., Prague, Videnska 1083, 14220 Prague 4, Czech Republic; ^4^Section of Immunology and Infection, Institute of Medical Sciences, University of Aberdeen, Foresterhill, Aberdeen AB252ZD, UK; ^5^Immunology and Virology Program, Centre for Ophthalmology and Visual Science, The University of Western Australia, Crawley, WA 6009, Australia; ^6^Centre for Experimental Immunology, Lions Eye Institute, 2 Verdun Street, Nedlands, WA 6009, Australia

## Abstract

The microbiota is a crucial modulator of the immune system. Here, we evaluated how its absence or reduction modifies the inflammatory response in the murine model of experimental autoimmune uveoretinitis (EAU). We induced EAU in germ-free (GF) or conventionally housed (CV) mice and in CV mice treated with a combination of broad-spectrum antibiotics either from the day of EAU induction or from one week prior to induction of disease. The severity of the inflammation was assessed by fundus biomicroscopy or by histology, including immunohistology. The immunophenotyping of T cells in local and distant lymph nodes was performed by flow cytometry. We found that GF mice and mice where the microbiota was reduced one week before EAU induction were protected from severe autoimmune inflammation. GF mice had lower numbers of infiltrating macrophages and significantly less T cell infiltration in the retina than CV mice with EAU. GF mice also had reduced numbers of IFN-*γ* and IL-17-producing T cells and increased numbers of regulatory T cells in the eye-draining lymph nodes. These data suggest that the presence of microbiota during autoantigen recognition regulates the inflammatory response by influencing the adaptive immune response.

## 1. Introduction

Considerable effort has been made to understand mechanisms leading to the initiation of autoimmune diseases, and yet the reason for the loss of self-tolerance is still not fully clarified. One hypothesis is that infection triggers autoimmune disease in genetically predisposed individuals by cross-reactivity between self-antigen and foreign antigen due to similarity between foreign and self-antigenic epitopes [1]. A second proposal is that infections may also trigger autoimmune disease through “bystander” activation of autoreactive T cells, in which self-antigen released during tissue damage is presented by activated innate immune cells or B cells, for instance, during mycobacterial infection [2, 3].

Uveitis or intraocular inflammation is a sight-threatening condition, which affects mostly people of working age. Despite improved therapeutic possibilities 10% of patients become blind [4, 5]. Many cases of intraocular inflammation are directly caused by infections such as toxoplasmosis, viral infections, and other pathogens. However, in around 50% of cases the etiology remains unknown. Some cases may be associated with systemic diseases such as sarcoidosis, multiple sclerosis, and ankylosing spondylitis in which infections as triggers of autoimmunity are also implicated. Specific agents such as* Chlamydophila*, Human Herpes Virus 6, and Epstein-Barr virus are suggested in either the development or the progression of multiple sclerosis [6]. However, both for “idiopathic” uveitis and for uveitis associated with systemic disease, the search for infectious causes is frequently fruitless.

Accordingly, animal models of autoimmune diseases have been developed most of which utilize immunization with an evolutionarily conserved “autoantigen” together with one or more adjuvants which usually comprise a component of an infectious agent such as heat-killed mycobacteria and pertussis toxin [7]. Adjuvants are required in order to activate innate immune cells via pathogen recognition receptors which include at least four classes of receptors, particularly toll-like receptors and C-type lectins. The need for adjuvants in generating experimental models implicates infectious agents in the induction of autoimmune disease and in this context, there has been considerable interest in the role of the microbiome in modulating susceptibility to autoimmune diseases.

The explosion of research into the nature of the microbiome using molecular genetic techniques to type and classify microbiota has revealed that commensal organisms exist in specific sites or niches in the body and are specific for each site. Initial studies on the gut microbiome in the context of inflammatory bowel disease revealed the role of the gut microbiome in maintaining immunological homeostasis in situ, thereby reducing the risk of inflammatory bowel disease [8]. These studies have been extended to other autoimmune disease models and for this purpose the use of germ-free (GF) mice has been extremely valuable [9–13]. Interestingly, the susceptibility to experimental autoimmune disease varied with the type of the disease: for instance, experimental autoimmune encephalomyelitis induced by myelin oligodendrocyte glycoprotein was found to be less severe in GF mice [14], while diabetes mellitus in nonobese diabetic mice was more severe [15–17]. Caspi et al. [18] and Nakamura et al. [19] have previously reported in a surrogate model of GF mice that alteration of the gut microbiota by using a combination of orally administered broad-spectrum antibiotics modulates the severity of EAU. More recently work from the same laboratory has used an interphotoreceptor retinoid-binding protein- (IRBP-) transgenic mouse model of spontaneous uveitis in antibiotic-treated and GF mice to demonstrate suppression of EAU [20]. Although this work describes a spontaneous model of EAU, which is considered by some to be more clinically relevant than the complete Freund's adjuvant- (CFA-) induced model, the mice contain an artificially high number of IRBP-specific CD4 T cells (>20% in the IRBP-161H clone). However, in CV mice and in humans, the precursor frequency of any particular antigen-specific T cell is known to be very low, if not rare [21]. It is therefore possible that in the IRBP-transgenic spontaneous model of uveitis, the sheer weight of numbers of antigen-specific T cells predicates a greatly increased risk of microbial antigen-induced T cell receptor cross-reactivity, which would then permit entry of activated T cells into tissue sites and further activation via cognate antigen.

We have therefore investigated whether reduction in the gut microbiome, by either antibiotic use as previously reported or as found in GF mice, modifies EAU induction in CV mice in which T cell activation is induced with a mycobacterial adjuvant (CFA) and is dependent on innate immune cell activation and signaling via the C-type lectin, dectin-1 [22]. We report that the severity of EAU is markedly reduced in GF mice and in mice which have been pretreated with antibiotics to reduce microbial burden but not when the microbial burden is reduced after activation of T cells has been induced.

## 2. Materials and Methods

### 2.1. Animals

We used inbred male and female mice of the C57BL/6J strain (5 to 8 weeks old). Mice were housed either at the conventional animal facility of Department of Pharmacology, First Faculty of Medicine, Charles University in Prague, where untreated CV mice were compared with CV mice treated with broad-spectrum antibiotics, or in the Laboratory of Gnotobiology at the Institute of Microbiology Academy of Sciences, Czech Republic, Novy Hradek, where experiments comparing GF and CV mice were performed.

The mice were rederived into GF conditions using Caesarean section and bred in sterile Trexler-type plastic isolators for many generations. The bedding, food pellets, and water were sterilized by gamma irradiation (25 kGy) or autoclaving. The long-term colonies of GF mice were supplied with sterile water and food pellets, HD2 extruded diet (Fitmin, Czech Republic), ad libitum. The GF status of colonies was evaluated weekly as fecal samples and cotton swabs from the isolator interior were tested for the presence of aerobic and anaerobic bacteria, mold, and yeast. The CV mice, which served as CV controls at this facility, were fed with the same diet and regularly tested for the absence of potential mouse pathogens, including strains of* Helicobacter muridarum* and* H. hepaticus*, according to internationally recognized standards (FELASA).

The use of animals for these experiments was approved by the Commission for Animal Welfare of the First Faculty of Medicine of Charles University in Prague, Czech Republic, and the Ministry of Education, Youth and Sports and by the Animal Care and Use Committee of the Institute of Microbiology, Academy of Sciences of the Czech Republic, according to animal protection laws.

### 2.2. EAU Induction

EAU was induced by subcutaneous inoculation of IRBP peptide 500 *μ*g per mouse in complete Freund's adjuvant in conjunction with intraperitoneal application of pertussis toxin (PT) 0.6 *μ*g according to a standard protocol [23, 24]. In brief, IRBP peptide 1–20 (interphotoreceptor retinoid-binding protein, also called retinol-binding protein 3-precursor fragment [Homo sapiens] H2N-GPTHLFQPSLVLDMAKVLLD-OH, New England Peptide, Gardner, USA) dissolved in DMSO (dimethyl sulfoxide, Sigma-Aldrich, St. Louis, USA) was emulsified in ratio 1 : 1 with CFA (Difco, USA) and the solution was applied subcutaneously.

### 2.3. Antibiotic Treatment

To reduce the microbial load, the mice were treated with broad-spectrum antibiotics (mixture of 500 mg/L of metronidazole (B. Braun, Czech Republic) and ciprofloxacin 100 mg/L (Ciprinol, Krka, Czech Republic)) in the drinking water as previously described [25]. Metronidazole (nitroimidazole) has a limited spectrum of activity that encompasses various protozoans and most Gram-negative and Gram-positive anaerobic bacteria. Ciprofloxacin is a second-generation fluoroquinolone, with a spectrum of activity, which includes Gram-negative and Gram-positive bacterial pathogens. To establish the importance of the microbiota with respect to disease induction, we initiated treatment either one week prior to EAU induction or on the day of EAU induction. In both experimental schedules, the antibiotic treatment continued until the end of the experiment.

### 2.4. Clinical Evaluation

*In vivo* clinical examination (fundus biomicroscopy) was performed using the TEFI imaging system [26–28]. An additional +4.0 diopter lens between the camera and the otoscope was used. During the procedure, the mice were under general anesthesia (ketamine 80 mg/kg and xylazine 5 mg/kg (both Bioveta, Slovakia) intraperitoneally). The fundi were imaged through a dilated pupil (tropicamide, Unitropic 1% oph. gtt., Unimed Pharma, Slovakia) and phenylephrine (Neosynephrin-POS 10% oph. gtt., URSAPHARM, Czech Republic). The otoscope was applied to the cornea using eye gel carbomerum (Vidisic gel, Bausch and Lomb, Czech Republic). A single image of the posterior central fundus from each eye was taken, transferred to a computer for analysis.

The inflammation was graded as described previously; see Table 1 [28]. Retinal inflammatory changes were evaluated separately for the optic disc, retinal vessels, and retinal tissue changes from the central fundus (Table 1). The mean overall clinical inflammation grade was then averaged. All samples were evaluated by two experienced ophthalmologists (PSS, AK) and the discussed consensus of the two evaluations was used.

### 2.5. Histological Evaluation

The mice were sacrificed on day 35 and the eyes were enucleated and immediately immersed in Tissue-Tek® O.C.T. Compound*™* (Sakura Finetek USA, Inc., Torrance, CA, USA) and frozen in 2-methylbutane (Sigma-Aldrich, St. Louis, USA) in liquid nitrogen. The samples were stored at −70°C until sectioning to 7 *μ*m thick slices (at −19 to −21°C). Sections were taken from both eye peripheries and centrally through the optic nerve. The samples were cut with a cryostat (Leica CM 1850) and stained with hematoxylin and eosin. These samples were then evaluated by two experienced ophthalmologists and graded using a standardized scoring system as previously published [7, 29, 30] and modified by the authors (Table 2). Eyes with congenital defects, such as microphthalmia or cataract, have been excluded from evaluation, which led to odd numbers in some graphs.

### 2.6. Immunohistochemistry

The immunohistochemistry was performed on six randomly selected mice from each group. T-lymphocytes were detected using a three-step immunoperoxidase method with polyclonal rabbit anti-human CD3 (Dako Denmark A/S, Glostrup, Denmark) diluted 1 : 200 in PBS containing 1.5% normal goat serum. This antibody is cross-reactive with mouse antigens [31]. Visualization of primary antibody binding was performed using secondary biotinylated anti-rabbit antibody (Dako) and the VECTASTAIN Elite ABC kit standard (Vector Laboratories, USA).

Macrophages were detected using a three-step immunoperoxidase method with monoclonal rat anti-mouse F4/80 antibody (clone BM8, Abcam, Cambridge, UK) diluted 1 : 100 in PBS containing 1.5% normal goat serum. Visualization of primary antibody binding was performed using secondary biotinylated anti-rat antibody (Abcam) and the VECTASTAIN Elite ABC kit standard (Vector Laboratories, USA). Positive cells were counted in two sections per eye, one from periphery and one from the centre, to obtain quantitative data.

### 2.7. Immunophenotyping by Flow Cytometry

Mouse mesenteric and cervical lymph nodes were separately harvested, mashed into cell suspension, washed in complete RPMI medium, and filtered through a 70 *μ*m cell strainer. For detection of regulatory T cells, the cell suspensions were washed, labeled with Fixable Viability Dye (eBioscience), blocked with anti-CD16/CD32 antibody, stained for surface CD4 and CD25, fixed and permeabilized overnight with Fixation/Permeabilization buffer (eBioscience), and stained for intracellular FoxP3. To analyze intracellular cytokine production, cells (2 × 10^6^ cells/mL in complete RPMI) were incubated for 5 hours with 50 ng/mL PMA, 500 ng/mL ionomycin (both from Sigma-Aldrich), and 2 *μ*M Monensin (eBioscience). After the incubation, the cells were washed, labeled with a viability dye, blocked, stained for surface CD4, fixed, and permeabilized as described above. Next, the cells were stained for intracellular cytokines with antibodies against IFN-*γ*, IL-17, and TNF-*α*. The data were acquired on a FACSCalibur flow cytometer and analyzed with FlowJo software. The cytokines were analyzed while gating on viable CD4^+^ cells. All monoclonal antibodies were purchased from eBioscience (San Diego, USA).

### 2.8. Data Analysis

Data were analyzed using GraphPad Prism Version 6.04 for Windows (GraphPad Software, San Diego, CA, USA, http://www.graphpad.com/). Kruskal-Wallis and Mann-Whitney nonparametric tests were used to evaluate differences between the groups and *p* < 0.05 was considered significant.

## 3. Results

### 3.1. The Severity of EAU Is Reduced in GF Mice

**Figure 1:**
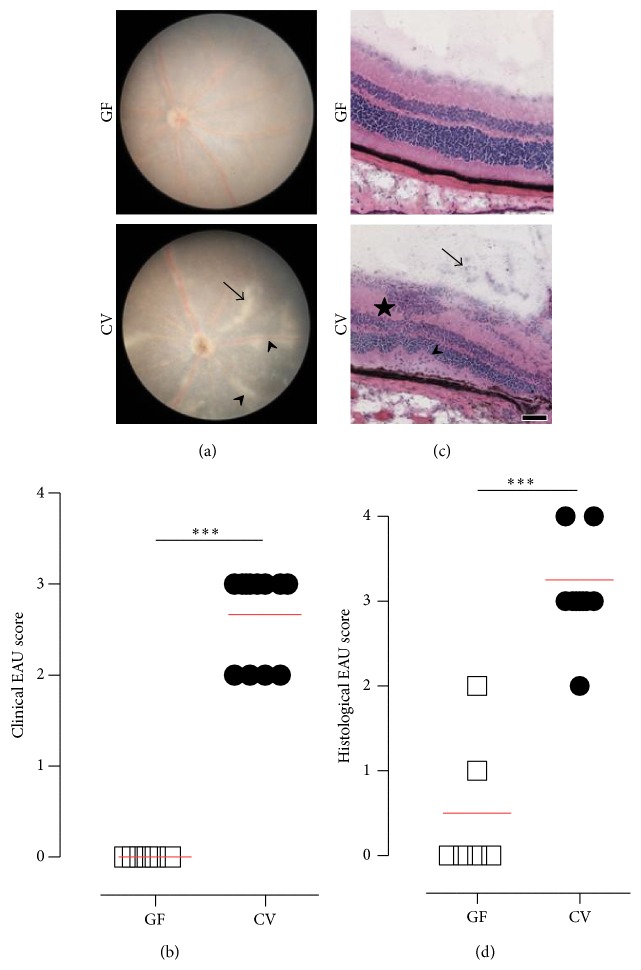
Severity of ocular inflammation in germ-free (GF) and conventional (CV) mice 35 days after EAU induction. (a) Representative photographs of retinal fundus of GF (grade 0) and CV (grade 2) mouse. The figure shows small and linear lesions (arrow), minimal optic disc inflammation, and moderate vascular cuffing (arrowheads). (b) Quantification of the clinical EAU score. By clinical fundoscopy, no inflammation was observed in the GF mice (3 animals) at day 35 after induction, whereas in control CV mice (6 animals), severe inflammation was observed. The red line in the graphs represents mean. ^*∗∗∗*^ *p* < 0.001 (Mann-Whitney test). (c) Representative microphotographs of hematoxylin and eosin-stained retina of GF and CV mice. The figure shows a large infiltrate (star) located in inner retinal layer, mild vitritis (arrow), and small retinal folds (arrowhead). (d) Quantification of histological EAU score. On histological evaluation, minimal to no signs of uveitis were observed in GF mice (6 animals) compared to severe uveitis in CV mice (6 animals). The red line in the graphs represents mean. ^*∗∗∗*^ *p* < 0.001 (Mann-Whitney test).

EAU was induced with sterile reagents in either GF or CV mice and the level of inflammation at day 35 by fundoscopy and histology was compared. EAU was significantly reduced in GF mice compared to CV controls. By clinical fundoscopy, no inflammation was observed in the GF mice at day 35 after induction, whereas in control CV mice, severe inflammation was observed as extensive signs of chorioretinal lesions, vascular sheathing (vasculitis), and vitreous haze (Table 1 and Figures 1(a) and 1(b); *p* < 0.001). On histological evaluation, minimal to no signs of uveitis were observed in GF mice compared to severe uveitis in CV mice (Table 2 and Figures 1(c) and 1(d); *p* < 0.001).

### 3.2. EAU in Mice Treated with Antibiotics

**Figure 2:**
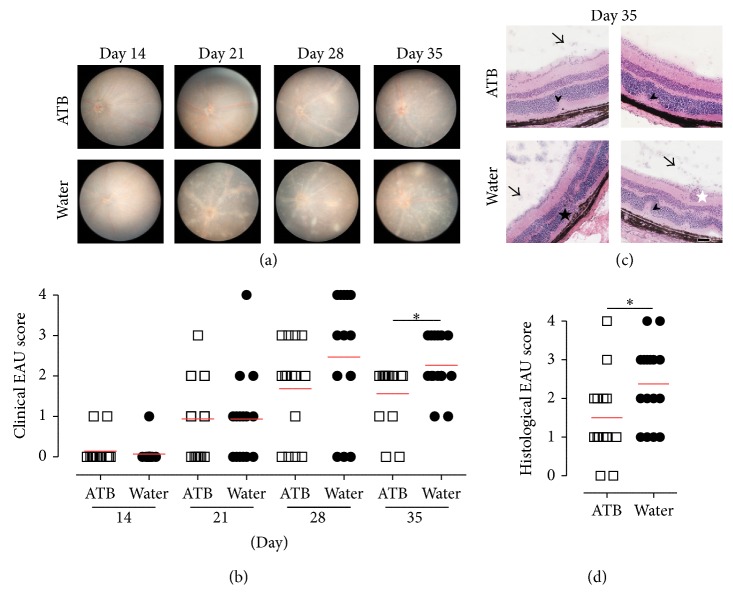
Reduced severity of EAU in mice treated with antibiotics (ATB) from one week before EAU induction (15 animals). Mice treated with metronidazole and ciprofloxacin commencing one week prior to EAU induction and continued for the course of the experiment had significantly lower levels of EAU compared to controls (15 animals) both clinically and histologically. (a) Representative photographs of retinal fundus at days 14, 21, 28 and day 35 after EAU induction show the development of ocular pathology in ATB and control mice. (b) Quantification of clinical EAU score. The red lines in the graphs represent mean. ^*∗*^ *p* < 0.05 (Mann-Whitney test). (c) Representative microphotographs of hematoxylin and eosin-stained retina of ATB-treated and control mice at day 35 after induction. Fewer signs of inflammation are present in ATB-treated compared to control mice, including cells in the vitreous (arrows) and small retinal folds (arrowheads), retinal neovascularization (black star), and vasculitis (white star). (d) Quantification of histological EAU score is shown. The red lines in the graphs represent mean. ^*∗*^ *p* < 0.05 (Mann-Whitney test).

**Figure 3:**
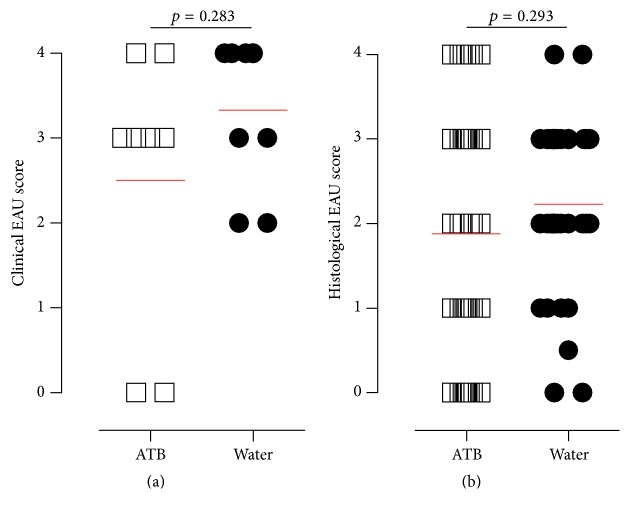
Antibiotics (ATB) administered from the day of EAU induction do not reduce the EAU severity. Quantification of (a) clinical and (b) histological EAU score at day 35 after induction is shown. The clinical data are from one of several independent experiments (ATB 11 mice, water 15 mice). The red lines in the graphs represent mean.

Since GF mice appeared to develop less severe EAU disease than CV housed mice, we speculated whether reduction in microbial load using antibiotic therapy would have the same effect as the GF state. We performed two experiments by administering antibiotics in CV mice either from the day of EAU induction or from one week before. Mice treated with metronidazole and ciprofloxacin (see Section 2) commencing one week prior to EAU induction and continued for the course of the experiment (a treatment which significantly reduces microbial burden [25]) had significantly lower levels of EAU compared to controls both clinically (Figures 2(a) and 2(b); maximal difference observed at day 35; *p* < 0.05) and histologically (Figures 2(c) and 2(d); *p* < 0.05). In contrast, mice treated with the same antibiotic regime but commencing on the day of immunization showed little difference in the level of EAU compared to controls (Figures 3(a) and 3(b)).

### 3.3. Immunohistology of the Eyes

**Figure 4:**
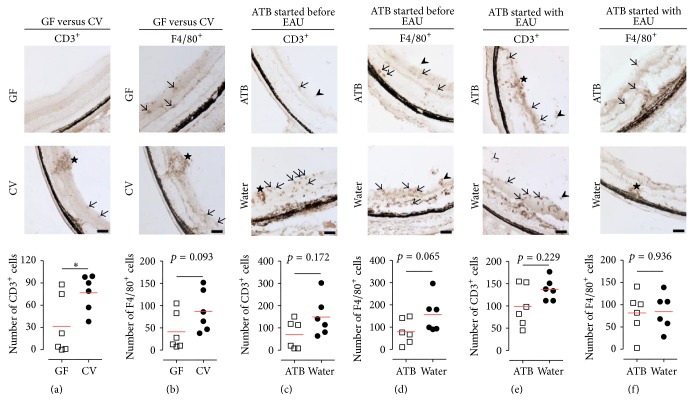
T cell and macrophage infiltration of the retina from germ-free (GF) mice (a, b), from mice treated with antibiotics (ATB) administered from one week (c, d), or from the day of EAU induction (e, f) and conventional (CV, water) mice 35 days after EAU induction. (a, c, e) CD3^+^ cells (T-lymphocytes) are shown both distributed as single cells in inner and outer retinal layers (arrows), in the vitreous (arrowheads) and concentrated as clumps in granulomas (stars). (b, d, f) F4/80^+^ cells (macrophages) are either present as single cells, in inner retinal layers (arrows), or accumulated in the periphery of granulomas (stars). Each point in the graphs shows the sum of all positive cells counted in two sections, one from periphery and one from the centre, from one randomly selected eye. The red lines in the graphs represent mean. ^*∗*^ *p* < 0.05 (Mann-Whitney test).

Immunohistological studies of the eyes performed on GF mice and littermate controls at day 35 (see Section 2) showed that qualitatively there was no difference in the nature of the cell infiltrate in the retina and choroid, which was composed of T cells and macrophages, distributed as individual cells or small cell aggregates (granulomas). However, quantitatively there was a significant reduction in CD3^+^ T cells (Figure 4(a); *p* < 0.05) and a similar but nonsignificant reduction in F4/80^+^ macrophages (Figure 4(b); *p* = 0.093) in the GF mice compared to the controls. Immunohistology was also performed on eyes of mice treated with metronidazole and ciprofloxacin from one week before or on the day of EAU induction (see Section 2; Figures 4(c), 4(d), 4(e), and 4(f)). Our data show that there was no significant qualitative or quantitative difference in the numbers and distribution of CD3^+^ T cells (Figures 4(c) and 4(e)) or F4/80^+^ macrophages (Figures 4(d) and 4(f)), when compared to controls.

### 3.4. Flow Cytometry of the Cervical and Mesenteric Lymph Nodes

**Figure 5:**
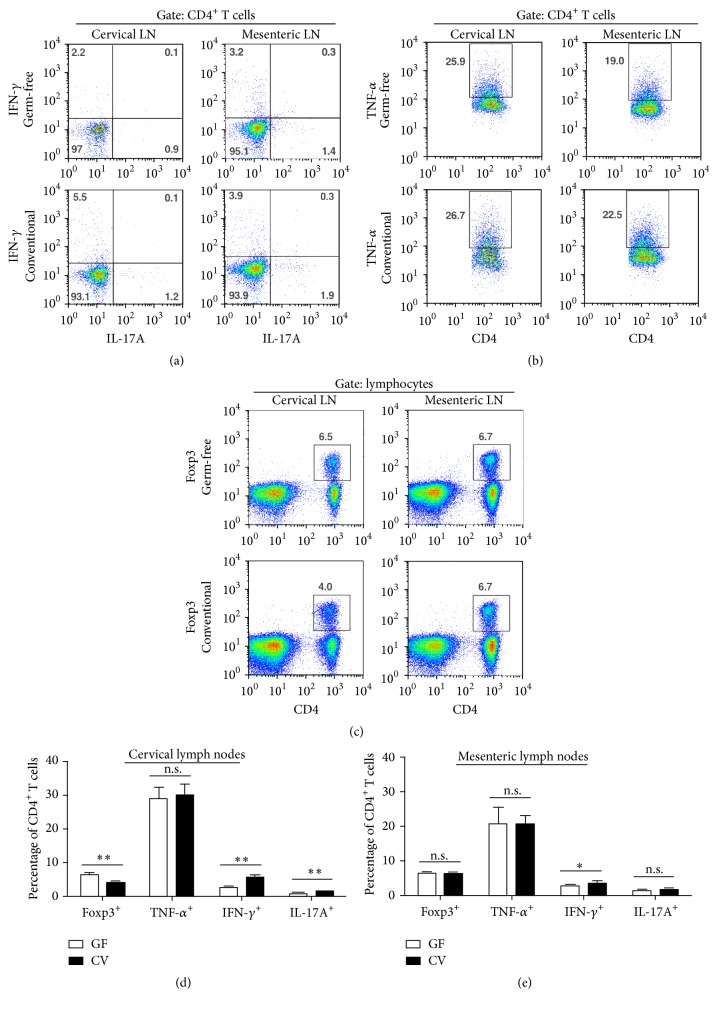
Flow cytometric analysis of lymphocyte populations in cervical and mesenteric lymph nodes. In the cervical lymph nodes of conventional (CV) mice, (a) the percentage of IFN-*γ* and IL-17-producing CD4^+^ T cells increased and (c) the percentage of regulatory Foxp3-expressing CD4^+^ T cells decreased compared to germ-free (GF) mice. In the mesenteric lymph nodes of CV mice, the environment was less proinflammatory showing only a small but significant increase of IFN-g-producing CD4^+^ T cells compared to GF mice. In both cervical and mesenteric lymph nodes, (b) the percentage of TNF-*α*-producing CD4^+^ T cells remained unchanged. The dot plots are representative of two independent experiments. The column graphs summarize the frequency of lymphocyte subpopulations in (d) the cervical and (e) the mesenteric lymph nodes. Each graph represents data from two independent experiments. ^*∗*^ *p* < 0.05, ^*∗∗*^ *p* < 0.01 (Mann-Whitney test). n.s. = not significant.

Since in CFA immunized mice, T cell activation occurs extraocularly with clonal expansion in the skin-draining lymph nodes beginning as early as 6 days after immunization [32] and subsequently in the eye-draining nodes as disease develops, we evaluated the phenotypes of lymph node cells by flow cytometry. In eye-draining cervical lymph nodes of CV mice, we observed an expansion of IFN-*γ*-producing (*p* < 0.01) and IL-17-producing CD4^+^ T cells (*p* < 0.01) and a reduced percentage of Foxp3^+^ Tregs (*p* < 0.01) in CV mice at day 35 after immunization with CFA and IRBP. However, the observed T cell expansion was considerably reduced in GF mice, which were similarly immunized. Interestingly, the percentage of CD4^+^TNF-*α* ^+^ T cells was similar in both CV and GF mice. Cell populations in non-eye-draining mesenteric lymph nodes showed a small increase in the percentage of IFN-*γ*-producing CD4^+^ T cells in CV mice (*p* < 0.05) which was significantly greater than IFN-*γ* ^+^ T cells in GF mice but there was no difference in IL-17-producing T cells in the mesenteric lymph nodes (Figure 5).

## 4. Discussion

The gut microbiota plays a significant role in the development of many inflammatory diseases, both in the gut [33, 34] and in distant organs [10, 35–38]. Continuous host-microbiota interactions determine the type and robustness of mucosal immune responses [39]. This is particularly important during the early stages of development, when the presence of microbiota is crucial for the maturation of the immune system in adult life [40]. As mentioned in Section 1, the presence of microbiota usually enhances inflammation in most animal models of colitis, multiple sclerosis, arthritis, or ankylosing spondylitis [13, 14, 41, 42], but it decreases the inflammation in models of type 1 diabetes [17].

Here, we show that the severity of the ocular inflammation in a murine model of autoimmune uveoretinitis is significantly lowered if the bacterial load is reduced either by rearing the mice in GF conditions (Figure 1) or by prophylactic treatment with broad-spectrum antibiotics (metronidazole and ciprofloxacin, Figure 2). Similar results were recently reported in the IRBP-transgenic mouse model of spontaneous uveitis [20]. However, the mode of uveitis pathogenesis in this model is dependent on a high peripheral precursor frequency of antigen-specific T cells (around 20% antigen-specific T cells are required in the periphery for expression of disease) which is far in excess of antigen-specific T cell precursor frequency in normal mice and humans [21]. Our data now show that in the standard model of EAU induced by IRBP in CFA, which more closely resembles human uveitis [43], GF mice have markedly reduced but not completely suppressed disease. We also show as has been reported previously [18, 19] that, by reducing the bacterial load by administration of broad-spectrum antibiotics, EAU is significantly reduced in severity. Importantly, in our study, when antibiotics were administered from the time of EAU induction, there was no significant effect on EAU severity. These experiments suggest that the preexisting microenvironment, particularly of the gut, has a significant role in determining the level of susceptibility to EAU and in addition that broad-spectrum antibiotic treatment may at least partially modify this environment, although not as effectively as in germ-free mice. This might be relevant for human medicine as treatment of uveitis patients with antibiotics in the past has not been limited only to infectious forms of uveitis [44]. The timing of the antibiotic treatment in patients, however, was always initiated after the development of immune response. In addition, some antibiotics, such as metronidazole, are known to suppress certain aspects of cell-mediated immunity, even when administered orally [45]. These experiments do not exclude the role of microbiota in the establishment of the immune response, since commensal microbes can produce molecules that regulate the immune system and these microbes may also be influenced by oral antibiotics [45–47]. In our experiments, when average water consumption and average mouse weight is calculated, the exposure to metronidazole was approximately 65 mg/kg for either gender of mice. Although this amount may have some minor suppressive effect on cellular immunity, the resistance of GF mice to EAU and the sensitivity of CV mice treated from the day of EAU induction suggest that it is more the effect on the microbiota than the immunosuppressive effect of metronidazole.

The surface of the eye, as for all mucosal surfaces, is colonized by microbes [48], and it is possible that the conjunctival microbiome may influence the development of ocular inflammation. However, such an event is unlikely unless there is a breach of the ocular surface barrier and any effect would likely be mediated through breakdown of the blood ocular barrier, in a similar manner that must apply to the gut microbiome. In this context, Horai et al. [20] have suggested that IL-17-producing antigen-specific T cells in the gut lamina propria are activated through their T cell receptor via noncognate microbial antigen derived from commensal bacteria. These activated cells then have the possibility to cross the blood retinal barrier and induce disease. In our experiments, T cells are a prominent infiltrating subset in the retina/choroid and both IFN-*γ*-producing (Th1) and IL-17-producing T cells are found in the eye-draining lymph node. However only a small induction of Th1 and no induction of Th17 cells was observed in the mesenteric lymph nodes draining the gut (Figure 5). In contrast, we have previously shown that T cell activation by CFA during EAU induction is mediated via dectin-1 [22], a known activator of IL-17-producing T cells, and that both Th1 and Th17 cells are involved. In addition, other innate receptors activated by mycobacterial proteins such as Mincle have been identified all of which appear to act via the CARD9 signaling complex [49]. We therefore propose, in the CFA model of EAU described here, that antigen-specific Th1 and Th17 cells are activated locally in the skin-draining lymph node from the immunization site, which then traffic through many tissues and sites including the gut, where the gut microbiome amplifies their activation status in a bystander fashion, rather than by inducing antigen-specific T cells via commensal microbial antigen.

Inflammation is a strictly compartmentalized process, although there is often some systemic reflection of this event. Therefore, we compared T cells in the eye, the local (cervical) and the distant (mesenteric) lymph nodes between GF and CV mice 35 days after the EAU induction. Using immunohistochemistry, we found that GF mice have significantly less CD3^+^ T cells and a similar but not significant reduction in F4/80^+^ macrophages in their eyes when compared with CV mice (Figure 4). In both groups, the localization of cells was similar, with T cells either clustered in granulomas or scattered in inner and outer retinal layers and macrophages located in the inner retinal layers. These data suggest that the lower eye infiltration with T cells in GF mice is the consequence and not the cause of the reduced level of EAU. The higher number or regulatory T cells in the cervical lymph nodes of GF mice, but not in mesenteric lymph nodes, also suggests that these cells are attracted to the local site of inflammation and may regulate the local immune response by bystander suppression.

## 5. Conclusions

In the current study, we show that the absence of microbiota or decrease of bacterial load before induction of inflammation significantly decreases the susceptibility of mice to EAU induced by IRBP in CFA. We show that reduction in the microbial burden induces changes in the strength of the T cell response in GF mice, with reduced T cell infiltration in the retina and also reduced Th1 and Th17-type T cell numbers in the eye-draining lymph node. This effect was not reiterated in antibiotic-treated mice suggesting that the reduction in the microbiota in antibiotic-treated mice was incomplete.

We propose that the presence of the microbiota promotes organ specific autoimmunity by amplifying the activation of antigen-specific T cells when these cells are induced in the secondary lymphoid organs as would occur in human disease. These results support the notion that the microbiota is important in pathogenesis of autoantigen-induced uveitis and that treatment with antibiotics may constitute an adjunct therapy for sight-threatening uveitis.

## Figures and Tables

**Table 1:** Clinical evaluation of retinal changes during EAU.

Features	Grade 1	Grade 2	Grade 3	Grade 4
Retinal tissue infiltrates	1–4 small lesions or 1 linear lesion	5–10 small lesions or 2-3 linear lesions	≥10 small lesions or ≥3 linear lesions	Linear lesions confluent
Optic disc	Minimal inflammation	Mild inflammation	Moderate inflammation	Severe inflammation
Retinal vessels	Engorged vessels with no perivascular cuffing	Engorged vessels and 1–4 mild cuffings	≥4 mild cuffings or 1–3 moderate cuffings	≥3 moderate cuffings or ≥1 severe cuffing

**Table 2:** Histopathological scoring system.

Features	Grade 0.5	Grade 1	Grade 2	Grade 3	Grade 4
Nongranulomatous infiltrate	Small in the ciliary body/retina/choroid	—	Cells in AC	—	Subretinal exudate
Vasculitis	—	occ./mild	≥2 vessels	≥10–50%	≥50%
Vitritis	—	Mild	Mild/moderate	Marked	Severe
Retinal folds	—	occ.	2	≥3	Extensive or detachment
Granulomas	—	—	1-2	≥3	≥3
Photoreceptor loss	—	—	Mild/moderate	Severe (≥60%)	Severe (≥60%)

## Abbreviations

CFA: Complete Freund's adjuvant
DMSO: Dimethyl sulfoxide
EAU: Experimental autoimmune uveoretinitis
IL: Interleukin
IRBP: Interphotoreceptor retinoid-binding protein
PBS: Phosphate-buffered saline
PT: Pertussis toxin
CV: Conventional controls
Th: T-helper lymphocyte.
